# 2,4,6-Trinitro­phenyl 4-chloro­benzoate

**DOI:** 10.1107/S1600536813007332

**Published:** 2013-03-23

**Authors:** Rodolfo Moreno-Fuquen, Fabricio Mosquera, Javier Ellena, Juan C. Tenorio, Carlos A. De Simone

**Affiliations:** a Departamento de Química – Facultad de Ciencias, Universidad del Valle, Apartado 25360, Santiago de Cali, Colombia; b Instituto de Física de São Carlos, IFSC, Universidade de São Paulo, USP, São Carlos, SP, Brazil

## Abstract

In the title benzoate derivative, C_13_H_6_ClN_3_O_8_, the planes of the benzene rings form a dihedral angle of 63.46 (5)°. The dihedral angles between the benzene ring and its nitro groups are 12.78 (16)° for the first *ortho*, 28.4 (4) and 17.4 (4)° for the second (disordered) *ortho* and 3.58 (16)° for the *para* nitro group. The central ester moiety, –C—(C=O)—O–, is essentially planar (r.m.s. deviation for all non-H atoms = 0.0229 Å) and forms dihedral angles of 7.37 (14)° with the chloro-substituted benzene ring and 69.85 (6)° with the trinitro-substituted benzene ring. One of the nitro groups was refined as disordered over two sets of sites with fixed site occupancies of 0.61 and 0.39. In the crystal, mol­ecules are linked by weak C—H⋯O hydrogen bonds, forming a three-dimensional network.

## Related literature

For the industrial and synthetic applications of nitroaryl compounds, see: Moreno-Fuquen *et al.* (2012*a* ▶) and references therein. For similar structures, see: Moreno-Fuquen *et al.* (2012*b* ▶,*c* ▶). For hydrogen bonding, see: Nardelli (1995 ▶). For hydrogen-bond motifs, see: Etter *et al.* (1990 ▶). For a description of the Cambridge Structural Database (CSD), see: Allen (2002 ▶).
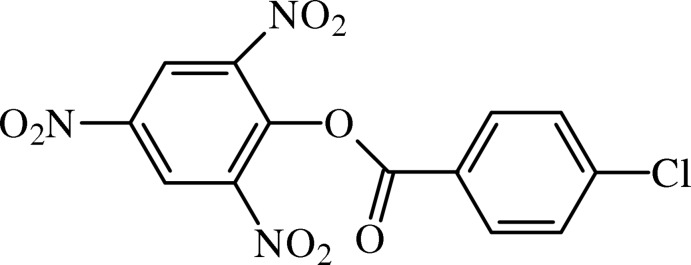


## Experimental

### 

#### Crystal data

C_13_H_6_ClN_3_O_8_
*M* *_r_* = 367.66
Monoclinic, 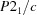

*a* = 9.3526 (3) Å
*b* = 11.4793 (3) Å
*c* = 13.6089 (4) Å
β = 93.612 (2)°
*V* = 1458.17 (7) Å^3^
*Z* = 4
Mo *K*α radiation
μ = 0.32 mm^−1^
*T* = 295 K
0.35 × 0.31 × 0.24 mm

#### Data collection

Nonius KappaCCD diffractometer
15908 measured reflections
3288 independent reflections
2424 reflections with *I* > 2σ(*I*)
*R*_int_ = 0.040

#### Refinement

*R*[*F* ^2^ > 2σ(*F* ^2^)] = 0.048
*wR*(*F* ^2^) = 0.151
*S* = 1.02
3288 reflections
246 parameters
H-atom parameters constrained
Δρ_max_ = 0.30 e Å^−3^
Δρ_min_ = −0.24 e Å^−3^

### 

Data collection: *COLLECT* (Nonius, 2000 ▶); cell refinement: *SCALEPACK* (Otwinowski & Minor, 1997 ▶); data reduction: *DENZO* (Otwinowski & Minor, 1997 ▶) and *SCALEPACK*; program(s) used to solve structure: *SHELXS97* (Sheldrick, 2008 ▶); program(s) used to refine structure: *SHELXL97* (Sheldrick, 2008 ▶); molecular graphics: *ORTEP-3 for Windows* (Farrugia, 2012 ▶) and *Mercury* (Macrae *et al.*, 2006 ▶); software used to prepare material for publication: *WinGX* (Farrugia, 2012 ▶).

## Supplementary Material

Crystal structure: contains datablock(s) I, global. DOI: 10.1107/S1600536813007332/lh5591sup1.cif

Structure factors: contains datablock(s) I. DOI: 10.1107/S1600536813007332/lh5591Isup2.hkl

Supplementary material file. DOI: 10.1107/S1600536813007332/lh5591Isup3.cml

Additional supplementary materials:  crystallographic information; 3D view; checkCIF report

## Figures and Tables

**Table 1:** Hydrogen-bond geometry (Å, °)

*D*—H⋯*A*	*D*—H	H⋯*A*	*D*⋯*A*	*D*—H⋯*A*
C13—H13⋯O4^i^	0.93	2.55	3.472 (3)	174
C5—H5⋯O8^ii^	0.93	2.53	3.457 (2)	174
C3—H3⋯O6*B* ^iii^	0.93	2.36	3.188 (5)	147
C12—H12⋯O1^iv^	0.93	2.51	3.377 (2)	156

Symmetry codes: (i) 
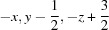
; (ii) 
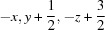
; (iii) 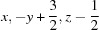; (iv) 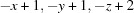.

**Fig. 1.**
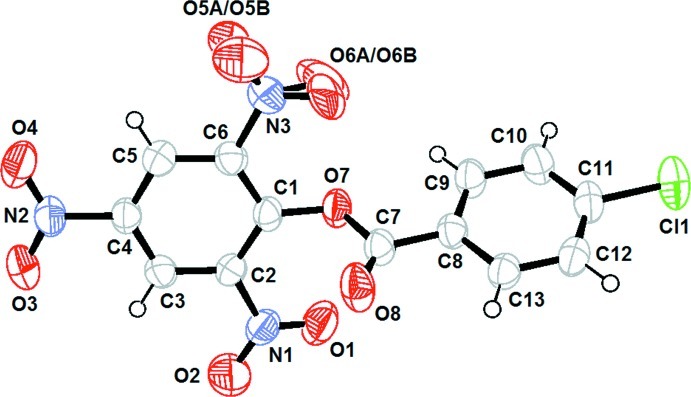
The molecular structure of the title compound with displacement ellipsoids drawn at the 50% probability level. H atoms are shown as spheres of arbitrary radius. The disorder of the O5/O6 atoms is shown.

**Fig. 2.**
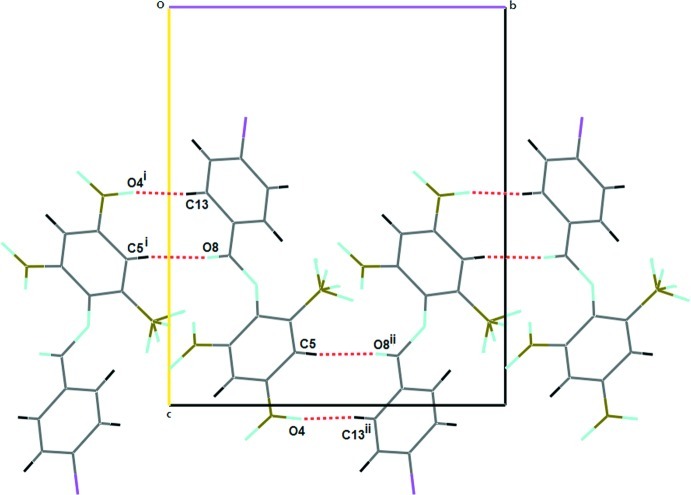
Part of the crystal structure of (I), showing chains formed by weak C—H···O hydrogen bonds (dashed lines) which run along [010]. Symmetry code: (i) -x,+y + 1/2,-z + 3/2; (ii) -x, y+1/2, -z+3/2.

**Fig. 3.**
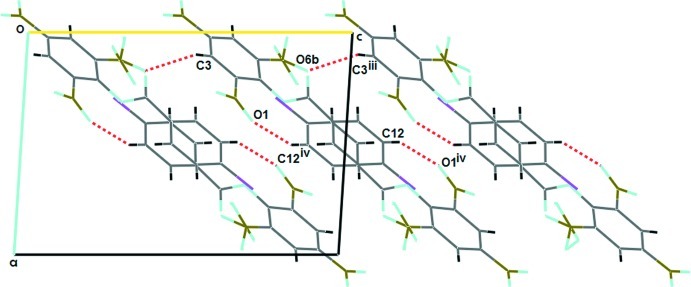
Part of the crystal structure of (I), showing chains formed by weak C—H···O hydrogen bonds (dashed lines) which run along [001]. Symmetry code: (iii) x,-y+3/2,+z-1/2; (iv) -x+1,-y+1,-z+2.

## supplementary crystallographic information

### Comment

The title compound (I) belongs to a group of molecules known as nitro aryl benzoates. The vast applications at the industrial and synthetic level of nitro aryl compounds have been described in an earlier paper (Moreno-Fuquen *et al.*, 2012*a*). Compound (I) is part of a series of studies on substituted 2,4,6-trinitrophenyl benzoates, also called picryl benzoates, undergone by our research group concerning the synthesis, properties and main features of the group of compounds. The molecular structure of (I) is shown in Fig. 1, with a numbering scheme similar to that for TNP3MeBA (Moreno-Fuquen *et al.*, 2012*a*), TNP4MeBA (Moreno-Fuquen *et al.*, 2012*b*) and TNPBA (Moreno-Fuquen *et al.*, 2012*c*) in order to simplify structural comparisons. The substituted picryl benzoates, including (I), show noticeable differences only in C1—O7 and C7—O7 bond distances, if they are compared with bond and angles parameters in other phenyl benzoates reported in the Cambridge Structural Database (Version 5.33, Allen, 2002). This fact has been highlighted in previous papers (Moreno-Fuquen *et al.*, 2012*b*,*c*) and it suggests a generalized effect over the ester moiety caused by the nitro substituents on the picryl fragment. The benzene rings of (I) form a dihedral angle of 63.46 (5)°. The central ester moiety forms an angle of 7.37 (14)° with the benzene ring to which it is attached. One of the nitro groups on the picryl fragment is disordered over two positions. The occupancies were initially refined but were fixed at 0.61 and 0.39 in the final cycles of refinement for O5A/O6A and O5B/O6B, respectively.

In the crystal, in a first substructure, the molecules are linked by weak C—H···O interactions, forming helical chains along [010]. The C5 atom of the phenyl ring at (*x*,*y*,*z*) acts as a hydrogen-bond donor to carbonyl atom O8 at (-*x*,+*y* + 1/2,-*z* + 3/2). Growth in this direction is reinforced by the weak C13—H13···O4 interaction, in which the C13 atom of the chloro substituted benzene ring at (x,y,z) acts as hydrogen-bond donor to atom O4 from one of the nitro groups at (-x, y-1/2, -z+3/2). The combination of these two contacts generate R^2^_2_(10) rings (Etter *et al.*, 1990), along [010] (See Fig. 2). This type of crystal growth for (I), was also observed for TNP3MeBA (Moreno-Fuquen *et al.*, 2012a). Additionally to those interactions, other weak C—H···O contacts were observed in (I) and they complement the main growth previously described. In a second substructure shown in Fig. 3, it can be observed the formation of dimers through the weak C12—-H12···O1 interactions. Indeed, the C12 atom at (x,y,z) acts as hydrogen-bond donor to O1 atom of the nitro group at (-x+1,-y+1,-z+2) forming R^2^_2_(20) rings (Etter *et al.*, 1990). These dimers are clearly connected to each other, through the weak C3—H3···O6B contact, allowing them to grow along [001]. The C3 atom at (x,y,z) acts as a hydrogen-bond donor to O6B atom of the nitro group at (x,-y+3/2,+z-1/2). Hence, in the crystal, the formation of an overall three-dimensional structure is observed, via weak C—H···O hydrogen bonds (see Table 1, Nardelli, 1995).

### Experimental

Reagents and solvents for the synthesis were obtained from the Aldrich Chemical Co., and were used without additional purification. The title molecule was obtained through a two-step reaction model. First the 4-chlorobenzoic acid (0.270 g, 0.734 mmol) was refluxed in an excess amount of thionyl chloride (10 ml) during an hour. Then thionyl chloride was distilled under reduce pressure to purify the 4-chlorobenzoyl chloride obtained as a pale yellow traslucent liquid. The same reaction flask was rearranged and a solution of picric acid (0.170 g, 0.734 mmol) in acetonitrile, was added dropwise with constant stirring. The reaction mixture was left to reflux for about an hour. A pale yellow solid was obtained after leaving the solvent to evaporate. The solid was washed with distilled water and cold methanol to eliminate impurities. Crystals of good quality and suitable for single-crystal X-ray diffraction were grown from acetonitrile. IR spectra were recorded on a FT—IR SHIMADZU IR-Affinity-1 spectrophotometer. Pale Yellow crystals; yield 72%; m.p 433 (1) K. IR (KBr) 3096.55 cm^-1^ (aromatic C—H); 1752.53 cm^-^1 (ester C=O); 1615.98, 1590.04 cm^-1^ (C=C); 1543.34 cm^-^1, 1340.73 cm^-1^ (–NO_2_); 1218.96 cm^-1^ (C(=O)—O).

### Refinement

All H-atoms were positioned at geometrically idealized positions with C—H distance of 0.93 Å and *U*_iso_(H) = 1.2 times *U*_eq_ of the C-atoms to which they were bonded.

### Crystal data

**Table:**

C_13_H_6_ClN_3_O_8_	*F*(000) = 744
*M**_r_* = 367.66	*D*_x_ = 1.675 Mg m^−^^3^
Monoclinic, *P*2_1_/*c*	Melting point: 433(1) K
Hall symbol: -P 2ybc	Mo *K*α radiation, λ = 0.71073 Å
*a* = 9.3526 (3) Å	Cell parameters from 7848 reflections
*b* = 11.4793 (3) Å	θ = 2.6–27.5°
*c* = 13.6089 (4) Å	µ = 0.32 mm^−^^1^
β = 93.612 (2)°	*T* = 295 K
*V* = 1458.17 (7) Å^3^	Block, pale-yellow
*Z* = 4	0.35 × 0.31 × 0.24 mm

### Data collection

**Table:**

Nonius KappaCCD diffractometer	2424 reflections with *I* > 2σ(*I*)
Radiation source: fine-focus sealed tube	*R*_int_ = 0.040
Graphite monochromator	θ_max_ = 27.5°, θ_min_ = 2.8°
CCD rotation images, thick slices scans	*h* = −12→12
15908 measured reflections	*k* = −14→14
3288 independent reflections	*l* = −17→17

### Refinement

**Table:**

Refinement on *F*^2^	Secondary atom site location: difference Fourier map
Least-squares matrix: full	Hydrogen site location: inferred from neighbouring sites
*R*[*F*^2^ > 2σ(*F*^2^)] = 0.048	H-atom parameters constrained
*wR*(*F*^2^) = 0.151	*w* = 1/[σ^2^(*F*_o_^2^) + (0.091*P*)^2^ + 0.2737*P*] where *P* = (*F*_o_^2^ + 2*F*_c_^2^)/3
*S* = 1.02	(Δ/σ)_max_ < 0.001
3288 reflections	Δρ_max_ = 0.30 e Å^−^^3^
246 parameters	Δρ_min_ = −0.24 e Å^−^^3^
0 restraints	Extinction correction: *SHELXL97* (Sheldrick, 2008), Fc^*^=kFc[1+0.001xFc^2^λ^3^/sin(2θ)]^-1/4^
Primary atom site location: structure-invariant direct methods	Extinction coefficient: 0.045 (6)

### Special details

**Table:**

Geometry. All esds (except the esd in the dihedral angle between two l.s. planes) are estimated using the full covariance matrix. The cell esds are taken into account individually in the estimation of esds in distances, angles and torsion angles; correlations between esds in cell parameters are only used when they are defined by crystal symmetry. An approximate (isotropic) treatment of cell esds is used for estimating esds involving l.s. planes.
Refinement. Refinement of *F*^2^ against ALL reflections. The weighted *R*-factor *wR* and goodness of fit *S* are based on *F*^2^, conventional *R*-factors *R* are based on *F*, with *F* set to zero for negative *F*^2^. The threshold expression of *F*^2^ > σ(*F*^2^) is used only for calculating *R*-factors(gt) *etc*. and is not relevant to the choice of reflections for refinement. *R*-factors based on *F*^2^ are statistically about twice as large as those based on *F*, and *R*- factors based on ALL data will be even larger.

### Fractional atomic coordinates and isotropic or equivalent isotropic displacement parameters (Å^2^)

**Table:**

	*x*	*y*	*z*	*U*_iso_*/*U*_eq_	Occ. (<1)
Cl1	0.70639 (6)	0.72855 (7)	1.22374 (4)	0.0773 (3)	
N1	0.2976 (2)	0.57696 (14)	0.65153 (12)	0.0561 (4)	
N2	−0.07414 (19)	0.80404 (16)	0.47344 (13)	0.0582 (4)	
N3	0.1244 (2)	0.95193 (16)	0.78955 (13)	0.0630 (5)	
O1	0.40062 (19)	0.57284 (15)	0.70877 (13)	0.0772 (5)	
O2	0.2690 (3)	0.50235 (18)	0.59209 (18)	0.1161 (9)	
O3	−0.0786 (2)	0.72668 (17)	0.41290 (15)	0.0935 (7)	
O4	−0.14293 (19)	0.89346 (16)	0.46545 (13)	0.0783 (5)	
O5A	0.0210 (6)	1.0035 (5)	0.7997 (4)	0.1039 (15)	0.61
O6A	0.2343 (8)	0.9689 (6)	0.8395 (6)	0.169 (3)	0.61
O5B	0.0649 (8)	1.0471 (5)	0.7629 (5)	0.0839 (18)	0.39
O6B	0.1736 (9)	0.9336 (6)	0.8683 (3)	0.083 (2)	0.39
O7	0.31371 (15)	0.75992 (11)	0.80241 (10)	0.0511 (3)	
O8	0.19504 (17)	0.61439 (15)	0.87062 (11)	0.0698 (5)	
C1	0.21381 (19)	0.76369 (15)	0.72449 (12)	0.0427 (4)	
C2	0.20321 (19)	0.67959 (15)	0.65074 (12)	0.0438 (4)	
C3	0.1074 (2)	0.69124 (15)	0.56973 (13)	0.0469 (4)	
H3	0.1000	0.6339	0.5214	0.056*	
C4	0.0233 (2)	0.78926 (15)	0.56216 (13)	0.0461 (4)	
C5	0.0276 (2)	0.87387 (16)	0.63325 (13)	0.0479 (4)	
H5	−0.0319	0.9387	0.6274	0.057*	
C6	0.1229 (2)	0.85976 (15)	0.71372 (12)	0.0455 (4)	
C7	0.2931 (2)	0.67990 (16)	0.87591 (12)	0.0457 (4)	
C8	0.40200 (19)	0.69270 (15)	0.95775 (12)	0.0435 (4)	
C9	0.5009 (2)	0.78335 (17)	0.96322 (14)	0.0522 (5)	
H9	0.5037	0.8366	0.9119	0.063*	
C10	0.5950 (2)	0.79402 (19)	1.04504 (15)	0.0574 (5)	
H10	0.6607	0.8549	1.0494	0.069*	
C11	0.5907 (2)	0.71382 (18)	1.12019 (14)	0.0528 (5)	
C12	0.4945 (2)	0.62241 (17)	1.11557 (13)	0.0513 (4)	
H12	0.4936	0.5687	1.1667	0.062*	
C13	0.3999 (2)	0.61173 (16)	1.03421 (12)	0.0469 (4)	
H13	0.3346	0.5505	1.0302	0.056*	

### Atomic displacement parameters (Å^2^)

**Table:**

	*U*^11^	*U*^22^	*U*^33^	*U*^12^	*U*^13^	*U*^23^
Cl1	0.0639 (4)	0.1147 (6)	0.0505 (3)	−0.0004 (3)	−0.0189 (2)	0.0022 (3)
N1	0.0678 (11)	0.0510 (9)	0.0490 (9)	0.0095 (7)	0.0005 (8)	0.0062 (7)
N2	0.0568 (10)	0.0640 (10)	0.0517 (9)	−0.0021 (8)	−0.0134 (7)	0.0040 (8)
N3	0.0814 (13)	0.0564 (10)	0.0505 (10)	0.0037 (9)	−0.0019 (9)	−0.0080 (8)
O1	0.0779 (11)	0.0792 (11)	0.0725 (11)	0.0261 (9)	−0.0106 (9)	0.0079 (8)
O2	0.150 (2)	0.0794 (12)	0.1119 (16)	0.0508 (13)	−0.0479 (14)	−0.0421 (12)
O3	0.1134 (16)	0.0845 (12)	0.0754 (12)	0.0096 (10)	−0.0499 (11)	−0.0188 (9)
O4	0.0763 (11)	0.0838 (11)	0.0717 (10)	0.0233 (9)	−0.0195 (8)	0.0077 (8)
O5A	0.119 (4)	0.093 (3)	0.100 (4)	0.039 (3)	0.012 (2)	−0.036 (3)
O6A	0.159 (6)	0.114 (4)	0.217 (8)	0.052 (4)	−0.115 (5)	−0.106 (5)
O5B	0.107 (5)	0.066 (4)	0.076 (4)	0.025 (3)	−0.022 (3)	−0.022 (3)
O6B	0.144 (6)	0.080 (4)	0.0251 (16)	−0.010 (3)	−0.008 (2)	−0.0062 (19)
O7	0.0551 (8)	0.0567 (7)	0.0399 (6)	−0.0104 (6)	−0.0098 (5)	0.0117 (5)
O8	0.0708 (10)	0.0877 (10)	0.0489 (8)	−0.0328 (8)	−0.0119 (6)	0.0194 (7)
C1	0.0458 (9)	0.0473 (9)	0.0344 (8)	−0.0055 (7)	−0.0011 (7)	0.0073 (7)
C2	0.0508 (10)	0.0414 (8)	0.0390 (8)	0.0001 (7)	0.0005 (7)	0.0060 (7)
C3	0.0569 (11)	0.0440 (9)	0.0394 (8)	−0.0039 (7)	−0.0013 (7)	−0.0003 (7)
C4	0.0469 (10)	0.0506 (10)	0.0398 (9)	−0.0033 (7)	−0.0052 (7)	0.0049 (7)
C5	0.0492 (10)	0.0487 (9)	0.0456 (9)	0.0030 (7)	0.0019 (7)	0.0045 (8)
C6	0.0532 (10)	0.0454 (9)	0.0379 (8)	−0.0015 (7)	0.0037 (7)	−0.0008 (7)
C7	0.0515 (10)	0.0521 (9)	0.0333 (8)	−0.0019 (8)	0.0018 (7)	0.0028 (7)
C8	0.0462 (9)	0.0497 (9)	0.0344 (8)	0.0027 (7)	0.0012 (7)	−0.0005 (7)
C9	0.0554 (11)	0.0614 (11)	0.0391 (9)	−0.0068 (8)	−0.0027 (8)	0.0059 (8)
C10	0.0570 (12)	0.0676 (12)	0.0465 (10)	−0.0103 (9)	−0.0055 (8)	−0.0004 (9)
C11	0.0468 (10)	0.0723 (12)	0.0384 (9)	0.0095 (9)	−0.0044 (7)	−0.0026 (8)
C12	0.0543 (11)	0.0598 (11)	0.0396 (9)	0.0112 (8)	0.0005 (7)	0.0073 (8)
C13	0.0510 (10)	0.0497 (9)	0.0399 (9)	0.0038 (7)	0.0019 (7)	0.0026 (7)

### Geometric parameters (Å, º)

**Table:**

Cl1—C11	1.7301 (19)	C2—C3	1.383 (2)
N1—O2	1.197 (2)	C3—C4	1.373 (3)
N1—O1	1.201 (2)	C3—H3	0.9300
N1—C2	1.472 (2)	C4—C5	1.370 (3)
N2—O3	1.210 (2)	C5—C6	1.377 (3)
N2—O4	1.213 (2)	C5—H5	0.9300
N2—C4	1.476 (2)	C7—C8	1.468 (2)
N3—O5A	1.149 (5)	C8—C9	1.391 (3)
N3—O6B	1.159 (6)	C8—C13	1.396 (2)
N3—O6A	1.212 (6)	C9—C10	1.380 (3)
N3—O5B	1.269 (7)	C9—H9	0.9300
N3—C6	1.477 (2)	C10—C11	1.379 (3)
O7—C1	1.369 (2)	C10—H10	0.9300
O7—C7	1.380 (2)	C11—C12	1.381 (3)
O8—C7	1.185 (2)	C12—C13	1.379 (3)
C1—C2	1.392 (2)	C12—H12	0.9300
C1—C6	1.395 (3)	C13—H13	0.9300
			
O2—N1—O1	123.13 (18)	C4—C5—C6	117.85 (17)
O2—N1—C2	117.33 (18)	C4—C5—H5	121.1
O1—N1—C2	119.51 (17)	C6—C5—H5	121.1
O3—N2—O4	124.39 (18)	C5—C6—C1	122.43 (16)
O3—N2—C4	117.75 (17)	C5—C6—N3	116.64 (16)
O4—N2—C4	117.86 (17)	C1—C6—N3	120.93 (17)
O5A—N3—O6B	105.7 (5)	O8—C7—O7	121.40 (16)
O5A—N3—O6A	122.7 (4)	O8—C7—C8	127.36 (16)
O6B—N3—O5B	124.2 (5)	O7—C7—C8	111.19 (15)
O6A—N3—O5B	111.3 (5)	C9—C8—C13	119.90 (17)
O5A—N3—C6	118.8 (3)	C9—C8—C7	122.85 (16)
O6B—N3—C6	120.1 (4)	C13—C8—C7	117.20 (16)
O6A—N3—C6	118.5 (3)	C10—C9—C8	119.82 (18)
O5B—N3—C6	115.6 (3)	C10—C9—H9	120.1
C1—O7—C7	117.57 (14)	C8—C9—H9	120.1
O7—C1—C2	123.42 (16)	C11—C10—C9	119.50 (19)
O7—C1—C6	119.22 (16)	C11—C10—H10	120.2
C2—C1—C6	117.23 (16)	C9—C10—H10	120.2
C3—C2—C1	121.39 (16)	C10—C11—C12	121.54 (17)
C3—C2—N1	116.22 (16)	C10—C11—Cl1	119.54 (16)
C1—C2—N1	122.31 (16)	C12—C11—Cl1	118.91 (15)
C4—C3—C2	118.59 (17)	C13—C12—C11	119.16 (17)
C4—C3—H3	120.7	C13—C12—H12	120.4
C2—C3—H3	120.7	C11—C12—H12	120.4
C5—C4—C3	122.47 (17)	C12—C13—C8	120.06 (18)
C5—C4—N2	119.06 (16)	C12—C13—H13	120.0
C3—C4—N2	118.47 (16)	C8—C13—H13	120.0
			
C7—O7—C1—C2	−74.2 (2)	C2—C1—C6—N3	178.57 (16)
C7—O7—C1—C6	109.99 (18)	O5A—N3—C6—C5	28.5 (4)
O7—C1—C2—C3	−175.31 (15)	O6B—N3—C6—C5	161.0 (5)
C6—C1—C2—C3	0.5 (2)	O6A—N3—C6—C5	−152.5 (5)
O7—C1—C2—N1	1.4 (3)	O5B—N3—C6—C5	−16.5 (5)
C6—C1—C2—N1	177.23 (15)	O5A—N3—C6—C1	−151.2 (4)
O2—N1—C2—C3	−12.3 (3)	O6B—N3—C6—C1	−18.8 (5)
O1—N1—C2—C3	165.66 (18)	O6A—N3—C6—C1	27.7 (6)
O2—N1—C2—C1	170.8 (2)	O5B—N3—C6—C1	163.7 (4)
O1—N1—C2—C1	−11.2 (3)	C1—O7—C7—O8	2.2 (3)
C1—C2—C3—C4	1.1 (3)	C1—O7—C7—C8	−175.40 (14)
N1—C2—C3—C4	−175.83 (16)	O8—C7—C8—C9	−170.4 (2)
C2—C3—C4—C5	−2.1 (3)	O7—C7—C8—C9	6.9 (3)
C2—C3—C4—N2	177.22 (16)	O8—C7—C8—C13	7.0 (3)
O3—N2—C4—C5	−178.2 (2)	O7—C7—C8—C13	−175.63 (15)
O4—N2—C4—C5	2.8 (3)	C13—C8—C9—C10	−1.1 (3)
O3—N2—C4—C3	2.4 (3)	C7—C8—C9—C10	176.21 (17)
O4—N2—C4—C3	−176.62 (19)	C8—C9—C10—C11	0.6 (3)
C3—C4—C5—C6	1.5 (3)	C9—C10—C11—C12	0.2 (3)
N2—C4—C5—C6	−177.86 (16)	C9—C10—C11—Cl1	−178.89 (16)
C4—C5—C6—C1	0.2 (3)	C10—C11—C12—C13	−0.5 (3)
C4—C5—C6—N3	−179.55 (17)	Cl1—C11—C12—C13	178.57 (14)
O7—C1—C6—C5	174.83 (15)	C11—C12—C13—C8	0.0 (3)
C2—C1—C6—C5	−1.2 (3)	C9—C8—C13—C12	0.8 (3)
O7—C1—C6—N3	−5.4 (2)	C7—C8—C13—C12	−176.69 (16)

### Hydrogen-bond geometry (Å, º)

**Table:**

*D*—H···*A*	*D*—H	H···*A*	*D*···*A*	*D*—H···*A*
C13—H13···O4^i^	0.93	2.55	3.472 (3)	174
C5—H5···O8^ii^	0.93	2.53	3.457 (2)	174
C3—H3···O6*B*^iii^	0.93	2.36	3.188 (5)	147
C12—H12···O1^iv^	0.93	2.51	3.377 (2)	156

Symmetry codes: (i) −*x*, *y*−1/2, −*z*+3/2; (ii) −*x*, *y*+1/2, −*z*+3/2; (iii) *x*, −*y*+3/2, *z*−1/2; (iv) −*x*+1, −*y*+1, −*z*+2.
